# Antitumor Effects and Mechanism of Novel Emodin Rhamnoside Derivatives against Human Cancer Cells *In Vitro*

**DOI:** 10.1371/journal.pone.0144781

**Published:** 2015-12-18

**Authors:** Jie-yu Xing, Gao-peng Song, Jun-peng Deng, Ling-zhi Jiang, Ping Xiong, Bin-jie Yang, Shan-shan Liu

**Affiliations:** 1 Department of Pharmaceutical Engineering, South China Agricultural University, Guangzhou, Guangdong, China; 2 Department of Biochemistry and Molecular Biology, Oklahoma State University, Stillwater, Oklahoma, United States of America; 3 College of Life Science, Shenzhen Key Laboratory of Microbial Genetic Engineering, Shenzhen University, Shenzhen, Guangdong, China; Yong Loo Lin School of Medicine, National University of Singapore, SINGAPORE

## Abstract

A series of novel anthracene L-rhamnopyranosides compounds were designed and synthesized and their anti-proliferative activities on cancer cell lines were investigated. We found that one derivative S-8 (EM-d-Rha) strongly inhibited cell proliferation of a panel of different human cancer cell lines including A549, HepG2, OVCAR-3, HeLa and K562 and SGC-790 cell lines, and displayed IC50 values in low micro-molar ranges, which are ten folds more effective than emodin. In addition, we found EM-d-Rha (3-(2”,3”-Di-O-acetyl-α-L-rhamnopyranosyl-(1→4)-2’,3’-di-*O*-acetyl-α-L-rhamnopyranosyl)-emodin) substantially induced cellular apoptosis of HepG2 and OVCAR-3 cells in the early growth stage. Furthermore, EM-d-Rha led to the decrease of mitochondrial transmembrane potential, and up-regulated the express of cells apoptosis factors in a concentration- and time-dependent manner. The results indicated the EM-d-Rha may inhibit the growth and proliferation of HepG2 cells through the pathway of apoptosis induction, and the possible molecular mechanism may due to the activation of intrinsic apoptotic signal pathway.

## Introduction

**Fig 1:**
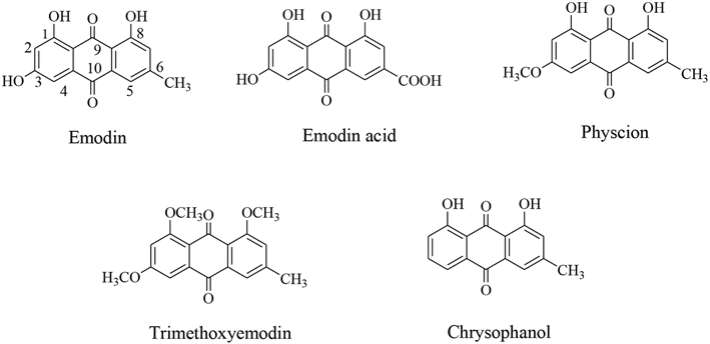
Chemical structures of emodin derivatives.

Emodin (3-methyl-1, 6, 8-trihydroxyanthraquinone) (Fig 1), a widely distributed anthraquinone, is derived from roots and rhizomes of *Rheum palmatum L*., and other plants such as *polygonum*, *Rhamnaceae*, *Leguminosae*, and *Liliaceae*. Emodin is an important component of Chinese herbs and structurally similar to anthracycline. It has the same tricyclic planar chromophore skeleton as certain antitumor antibiotics, such as daunorubicin and mitoxantrone which can intercalate DNA of cancer cells. The antitumor activity of emodin has been well documented. It was demonstrated that emodin possess antiproliferation, preventing cancer metastasis[1–3], sensitizing cancer cells to radiotherapy and chemotherapeutic agents [4–6], anti-angiogenic [7, 8] and reversing multidrug resistant(MDR) of cancer cells [9]. It has been confirmed that emodin is a broad-spectrum inhibitory agent of cancer cells, including leukemia [10, 11], lung cancer [12–14], human tongue squamous cancer [15, 16], colon cancer [17, 18], gallbladder cancer [19–21], pancreatic cancer [22–24], breast cancer [25–27], human cervical cancer [28] and hepatic carcinoma cells [29–31]. The anticancer mechanisms of emodin were involved in many biological pathways [32–34], such as casein kinase Ⅱand ERK1/2. However, emodin is an unsatisfying chemotherapeutic agent for cancer due to its deficiency in bioactivity, relatively poor bioavailability and toxicity in vivo. Despite this, emodin, as a natural compound, provide excellent basis for developing novel chemoprevention and chemotherapeutic agents against cancers (Fig 1). Since potential candidates from natural products were considered as one of the most productive strategies in current drug discovery and development [35].

**Fig 2:**
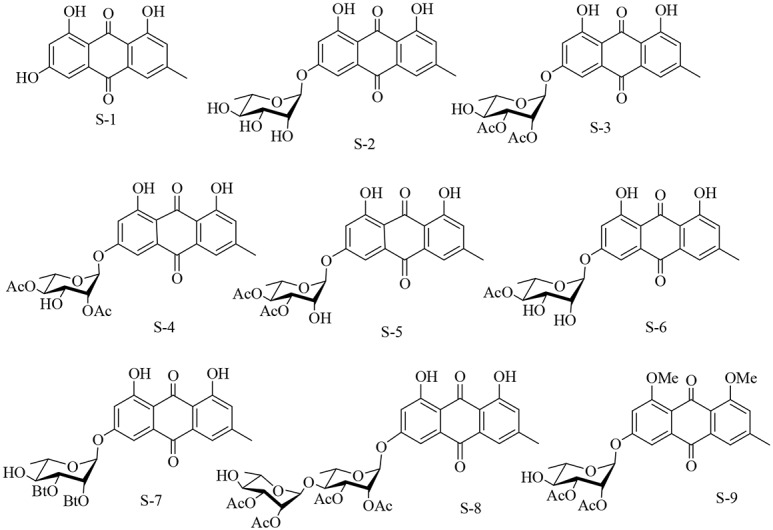
Chemical structures of emodin rhamnopyranosides derivatives.

Up to now, structural modification of emodin mainly involved the transformation of the side chain, including methyl, hydroxyl and aryl ring portion. In fact, it was shown that the introduction of side chains such as polymethyleneamine, sugar or heterocycle to emodin may enhanced anti-tumor activity [36–38]. In addition, the other achieved derivatives through introducing amino groups and glycosidic bonds also showed higher anticancer activity [39–41]. Emodin glycoside derivative have been isolated from *R*. *nepalensis*, *Rhamnaceae* plants [42], *Rhamnus frangula L*. [43] and *Rumex japonicus Houtt*.[44]. Some natural emodin glycoside derivatives, such as *frangulin B* and *2*,*3-di-O-acetylfrangulin A*[45–47], showed significantly higher antitumor activity than emodin. These studies demonstrated that addition of sugar chains at the C3-OH site on emodin molecule not only increased its solubility but also significantly improved its anti-tumor activity [46]. So far, however, the research on emodin derivatives of glycosylation modifications is rarely reported. Recently, we have synthesized a series of novel anthracene L-rhamnopyranosides derivatives of emodin by connecting L-rhamnopyranosides to a planar aromatic molecule (Fig 2) [48]. In this study, we screened and analyzed the antitumor effects of all derivatives. We found one compound, EM-d-Rha, inhibit strongly the growth and proliferation of cancer cells, which were almost ten folds stronger than emodin. In this study, we further demonstrated the antiproliferative activity of EM-d-Rha on cancer cell, and explored the mechanism action of EM-d-Rha inhibit the growth and proliferation of HepG2 cells.

## Materials and Methods

### Emodin derivatives synthesized

The synthesis of target compounds S-2, S-3, S-4, S-5, S-6, S-7, S-8, S-9 was the same as described previously (Fig 2) [48].

### Cell lines and chemical reagents

Human lung cancer A549, Human hepatic carcinoma HepG2, Human breast cancer MCF-7, Human prostate cancer PC-3, Human cervical cancer HeLa, Human chronic myeloid leukemia K562, Human gastric cancer SCG-7901, Madin-Darby Canine Kidney cell(MDCK), Human normal liver cell L02 and Human ovarian cancer OVCAR-3 cells were obtained from the American Type Culture Collection (Manassas, VA). All except leukemia K562 cells were cultured at 37°C in a humidity controlled atmosphere containing 5% CO2 in Dulbecco’s Modified Eagle Medium supplemented with sodium bicarbonate (2.2%, w/v), L-glutamine (0.03%, w/v), penicillin(100 μg/mL), streptomycin (100 μg/mL), and fetal Bovine serum (FBS, 10%). Leukemia K562 cells were cultured in RPMI-1640 complete medium supplemented with 10% FBS.

Cisplatin (*TOKYO Chemical Industry Co*. *LTD*), HCPT (*SHAANXI Sciphar Natural Products Co*.*Ltd*), Dimethyl Sulphoxide (DMSO, *MP Biomedicals LLC*), Fetal Bovine Serum(FBS, Hyclone, USA), Dulbecco’s Modified Eagle Medium(DMEM, *Hyclone*, *USA*), RPMI Media 1640 (*Hyclone*, *USA*), Penicillin-Streptomycin Double antibiotic (*SIGMA-ALDRICH*), 3-(4,5-Dimethylthiazol-2-yl)-2,5-Diphenyltetrazolium Bromide (MTT, *SIGMA-ALDRICH*), 0.25% Trypsin-EDTA (*Gibco*, *USA*), Trypan Blue Stain (*SIGMA-ALDRICH*), Hoechst 33342 Staining Solution (*Pik days Institute of Biotechnology*, *JiangSu*), Apoptosis-DNA ladder Extraction kit (*Pik days Institute of Biotechnology*, *JiangSu*), Annexin Apoptosis Detection Kit V-F1IC/7-AAD (*BD Pharmagen Company*, *USA*), PI/RNase Staining Solution (*BD Pharmagen Company*, *USA*), E.Z.N.A^TM^ Total RNA Kit II (*OMEGA*), M-MLV Reverase Transcriptase (*Promega*), dNTP (*Promega*), Oligo (*Promega*), GoTaq®qPCR Master Mix (*Promega*).

### Cell proliferation and cell viability assay

The viability of cancer cells was evaluated by MTT assay. Briefly, cells were inoculated in 96-well culture plates at the density of 1×10^4^/well with complete medium containing 10% fetal calf serum in a final volume of 0.2 mL. Cells were incubated at 37°C for at least 24 hours to allow optimal attachment. When the cells reached 60% confluence, they were treated with Cisplatin, HCPT and emodin rhamnopyranosides derivatives in various concentrations, and incubated in a humidity-controlled 5%CO_2_ incubator at 37°C for 72 hours. Medium were exchanged once every 24 hours. Cell control group and blank group were set up in the same manner, with each group having six parallel wells. Cell survival was assessed by directly adding 22 μL of 5mg/mL MTT to 0.2 mL medium. After three hours, the formazan precipitate was dissolved in 200μL isopropanol per well, and completely mixed under room temperature. Afterwards, the optical density of each well was detected at 570 nm wavelength by iMark microplate reader (*Bio-rad*,*USA*). This experiment was repeated at least three times.

### Morphological analysis

For morphological observation, HepG2 cells in logarithmic growth phase were seeded in a 96-well plate at the density of 1×10^4^/well with 0.2 mL complete medium containing 10% fetal calf serum. Cells were allowed to attach at 37°C for 24 hours. Then cells were treated with EM-d-Rha and HCPT at a specific concentration. Each group has six parallel wells. Cells were incubated in a humidified 5%CO2 incubator at 37°C for 72 hours. Medium were exchanged once every 24 hours. After 48hrs, the growth states and morphological changes of HepG2 cells were observed with an inverted microscope (*Nikon*, *Japan*).

### Fluorescence microscopy assay

HepG_2_ cells were inoculated in duplicate at 3×10^5^/well density in 6-well plate, and allowed to attach at 37°C for 24 hours. Afterwards, cells were treated with 10 μM HCPT, 25 μM emodin and specific concentration EM-d-Rha (0.1μM, 0.5μM, 2.5μM), respectively. Cell control group was set in the same way. Then cells were incubated in a humidified incubator with 5% CO_2_ at 37°C for 72 hours. Medium were exchanged as described previously. In the end, all medium were discarded and cells were digested with 2mL 0.25% Trypsin-EDTA solution at 37°C for 5 min. After cells were harvested, cells were washed with PBS twice followed by centrifuging at 300×g for 5 minutes. Finally, cells were re-suspended in 100μL cold PBS after the supernatant was discarded. Cells were cultured in the dark for 10 minutes at room temperature after adding 100μL 1mg/mL Hoechst 33342 staining solution. Cells were harvested again as described previously to discard the staining solution. Wash the harvested cells twice with PBS before applying to the clean glass slides. The morphological changes of cells nuclei were observed and photographed under the fluorescence microscope (*Olympus*, *Japan*) at 350 nm excitation wavelength. The experiment was repeated three times.

### DNA ladder assay

DNA ladder formation is an important criterion to determine cells apoptosis. To confirm the apoptosis induction effect, the analysis of DNA fragmentation was conducted. HepG2 cells in logarithmic growth phase were inoculated in 10cm diameter culture plates at a density of 5×10^5^ cells with 12 mL complete medium containing 10% fetal calf serum, and allowed to attach at 37°C for 24 hours. Afterwards, cells were treated with EM-d-Rha and Cisplatin at a specific concentration. Cells were cultured and harvested as described previously. Finally, DNA of all samples was extracted using the apoptosis DNA ladder detection kit following the manufacturer’s instructions. The DNA fragmentation was assayed by electrophoresis on a 1.5% agarose gel containing 0.5mg/mL ethidium bromide (EtBr) at 5V/cm for 2.5hours and photographed under ultraviolet. The size of the DNA fragmentation was compared with the marker (molecular weight standard).

### Flow cytometry analysis

Flow cytometry analysis was determined using Annexin V-APC/7-AAD Apoptosis Detection Kit (*BD Parmagen*, *U*.*S*.*A*). HepG_2_ cells were inoculated at a density of 3×10^5^/well in 6-well plates, and allowed to attach at 37°C for 24 hours. Afterwards, cells were treated with specific concentration EM-d-Rha, and cultured and harvested as described previously. Cells of each sample were resuspended in 300μL cold binding buffer before adding 2.5μL Annexin V-APC conjugate and 5μL 7-AAD solution. Then cells were incubated for 15 min on ice in the dark. Cell suspension was diluted to a final volume of 250μL with ice-cold, 1× Annexin V Binding Buffer. The assay was conducted by flow cytometry (*BD FACSCalibur*,*USA*). Scatter plots were generated using Cell Quest pro software (*BD Biosciences*, *San Jose*, *CA*). The experiment was repeated three times.

### Analysis of mitochondrial membrane potential

HepG_2_ cells were inoculated in 10cm diameter culture plates at a density of 5×10^5^ cells, and allowed to attach at 37°C for 24 hours. Afterwards, cells were treated with 5.0μM EM-d-Rha for 0, 24h, 48h and 72h respectively. Cells were incubated in a humidified incubator supplied with 5% CO_2_ at 37°C for 72 hours. Medium were exchanged once every 24 hours. The harvested cells of each sample were suspended in 500μL PBS before adding 5 μL Rhodamine123. Then samples were incubated at 37°C in the dark for 15 min. Cells were again collected by centrifugation at 300×g for 5 minutes and the supernatant was discarded. The cell pellets were washed twice with 4°C PBS. Finally cells of all samples were resuspended in 500μL PBS. Before performing assay, adjusting excitation wavelength and emission wavelength to 480nm, 520nm respectively, the ΔΨm changes of HepG2 mitochondrial were measured with a flow cytometer (*BD FACSCalibur*, *BD Biosciences*, *US*). The experiment was repeated at least three times.

### Cell cycle analysis (PI staining)

HepG2 cells in logarithmic growth phase were inoculated in 10cm culture plates at density of 5×10^5^ cells and incubated overnight and allowed to attach. After being exposed to EM-d-Rha at specific concentrations for 48h, cells were harvested and washed with cold PBS twice. Then the collected cells were fixed with 1mL PBS and 3mL cold 100% ethanol at 4°C overnight. After that, the cells were again harvested by centrifugation at 300×g for 10 min. The cells were washed with cold PBS and re-suspended in 450 μL PBS, and then treated with 50μL ribonuclease (RNase,10mg/mL) at 37°C for 15min. 500μL of propidium iodide (PI, 50μg/ml) was added to the samples. After the cells were incubated for 30 min at 4°C in the dark, the cell cycle distribution was measured using an BD FACSCalibur Flow cytometer (*BD Biosciences*, *USA*), and 10,000cells were counted for each sample. The percentage of cells in different phases of the cell cycle was analyzed by flow Jo (*BD Biosciences*, *USA*). The experiment was repeated three times.

### Gene analysis by RT-qPCR

HepG_2_ cells were inoculated in 6-well culture plates at a density of 3×10^5^ cells/well, and allowed to attach at 37°C for 24 hours. Then cells were treated with specific concentrations of EM-d-Rha and incubated in a humidified incubator supplied with 5% CO_2_ at 37°C for 48 hours. Medium were changed once every 24 hours. Afterwards, mRNA was extracted from every group using E.Z.N.A.^TM^ Total RNA Kit II according to the manufacturer’s instructions. The extracted mRNA was dissolved in 20 μL ddH_2_O (*Qiagen*) containing diethyl pyrocarbonate (DEPC). The concentration of mRNA was quantified with a NanoDrop ND-100 device (Thermo Fisher Scientific). cDNA was synthesized using 3μg of the extracted mRNA from each sample with PCR system (*BIO-RAD*,*USA*). The cDNA synthesis reaction was prepared according to the manufacturer’s instructions by mixing 3μg mRNA, 4.0μL 5× MMLV buffer, 3 μL oligodT18, 0.5μL 10 mM dNTP solution, 0.8μL RNase inhibitor, 3.7μL MMLV Rtase, and 6.0μL DEPC ddH_2_O in PCR tubes. The reaction condition was 5min at 70°C, 60 min at 42°C and 10min at 4°C. The products were used for subsequent RT-qPCR analysis. The level of mRNA expression was measured by RT-qPCR using a CFX96^TM^ C1000 Real-Time PCR System (*BIO-RAD*, *USA*).The primers were designed using idt.dna software system (http //www.idtdna.com). The specific primers were: Cyto C: 5′-AGATGGTGAGCACAAGGTAAG-3′ (forward), 5′- CTCACTGTCCCACAAAGATACA -3′ (reverse); Caspase 3: 5′- CCTACAGCCCATTTC TCCATAC-3′ (forward), 5′-GCCTCACCACCTTTAGAACAT-′ (reverse); β-actin: 5′-GGACCTGACTGACTACCTCAT-3′ (forward), 5′-CGTAGCACAGCTTCTCCTTAAT-3′ (reverse). The product sizes of Cyto C, Caspase 3 and β-actin were 91bp, 125bp and 107bp, respectively. The PCR reaction was performed in a final volume of 20μL using an optical 96-well tray. The reaction mixture consisted of 10μL of SYBR I green Mix/ GoTaq®qPCR

Master Mix (*Promega*), 0.3μL of each primer (10μM), and 1.0 μL cDNA template. PCR cycling condition was one cycle of 5min at 95°C, 39 cycles and each of which was consisted of 15 seconds at 95°C, 15 seconds at 62°C and 30 seconds at 72°C, with final extension at 60°C for 5 Seconds. The thermal melt parameter was investigated to assess the homogeneity of PCR. Each sample was analyzed in quadruplicate and mean was used for further analysis. The mRNA expression levels of each sample were quantified by measuring the threshold cycle (CT) values of genes. The relative values of the target gene expression were calculated according to CT values of the target genes and the reference gene (β-actin) by applying Livak and Schmittgen method. That is, it equals to 2^-Δ ΔCT^, ΔCT equals the mean of Ct for target gene minus the mean of Ct for β-actin gene, and ΔΔCT equals ΔCT of the treated group minus ΔCT of the control group.

### Western blot analysis

HepG_2_ cells were inoculated, cultured and harvested as described previously. Afterwards, the experiment was performed on ice. The harvested cell pellet was washed with cold PBS before being resuspended in 300μL cytosol lysis buffer through vortex. Then cells of all samples were lysed by sonication method on the ice using the ultrasonic cell disruptor (100 W for 30s×4 times). Total protein of each sample was measured by Bradford assay using protein quantification reagents from Bio-Rad. All samples were added 2×loading buffer before analyzing on 12% gels by SDS-PAGE. Then the proteins were transferred to nitrocellulose membranes by electro-blotting. The membranes were blocked in 1% bovine serum albumin (BSA) or 5% milk solution at room temperature for 30min, followed by incubation with the primary antibody against rabbit (*Univ-bio*, *China*) at 4°C overnight. The membranes were then washed three times with 1×TBST, each time last for 5 mins. The membranes were then incubated with the secondary antibodies for 3 h at 4°C, followed by washing with 1×TBST as described previously. Finally, the immuno-reactive bands were detected and documented using an enhanced chemiluminescent Western blotting system (*Thermo-scientific*, *USA)* on a Versa Doc imaging system.

### Statistical analysis

All data were presented as Mean ± S.E.M. The SPSS statistical Software for Windows Version 17.0 was used for one-way ANOVA analysis followed by the Tukey HSD test for multiple comparisons when appropriate. Differences in measured variables between two groups were compared by Student’s t-test. P < 0.05 was considered to be statistically significant. GraphPad Prism 5 software (*GraphPad Software*, *Inc*, *San Diego*, *CA*, *USA*) was used for the analysis. For RT-qPCR and flow cytometry experiments, data were representative of at least three independent experiments.

## Results

### Cell viability assay

We have tested the effects of various concentrations emodin (S-1) and its derivatives (S-2, S-3, S-4, S-5, S-6, S-7, S-8, S-9) on the viabilities of the tested human cancer cells lines(Fig 2 and Table 1). So the half-inhibitory concentration (IC_50_) values were calculated from these data. The data indicated that different derivatives inhibit the growth of different cell lines with varying degree (Table 1).

**Table 1:** IC50 values of emodin and its derivatives in different cell lines.

Compounds	IC50 values (μg/mL)				
	A549	HepG2	OVCAR-3	HeLa	MDCK
S-1	19.54	12.79	25.82	12.14	5.81
S-2	>20.00	>20.00	>20.00	>20.00	>20.00
S-3	>20.00	>20.00	>20.00	>20.00	>20.00
S-4	9.27	>20.00	>20.00	>20.00	>20.00
S-5	3.16	1.32	>20.00	5.34	14.8
S-6	2.25	>20.00	>20.00	>20.00	21.1
S-7	2.78	11.96	21.94	3.32	11.16
S-8	2.60	0.65	0.61	1.15	18.50
S-9	3.63	4.43	9.72	4.93	1.39

According to the efficacy evaluation standards of antitumor drug in vitro tests, the IC_50_ values of the synthesized compound or plant extract should be less than 10μg/mL and the efficacy should has a dosage-dependent manner, meanwhile the maximum inhibitory effect should exceed 80%. If the tested compound meet all of the described criteria, it was considered to possess anti-proliferation and inhibiting growth effects on cancer cells in vitro.

As shown in Table 1, among all the synthesized compounds, S-8 and S-9 derivatives showed stronger inhibitory effect on the tested cancer cells than parent emodin. Actually, S-8 act better than S-9. When compared to the IC_50_ of emodin, the IC_50_ values of S-8 on A549, HepG2, OVCAR-3 and HeLa cells decreased by 7.52, 19.68, 42.3 and 10.56 fold, respectively. In another words, S-8 enhanced anticancer activity by roughly ten orders of magnitude. Notably, HepG2 and OVCAR-3 cells were found to be susceptible to S-8 derivative.

### Structure-activity analysis of emodin rhamnopyranosides derivatives

Cell viability assay proved that rhamnopyranosides derivatives acted better than parent emodin. But it remains unclear how the altered chemical structures contributed to enhanced activity. So we preliminary analyzed the anticancer structure-activity relationship (Fig 2 and Table 1). From the in vitro data, it could be seen that the introduction of rhamnose can help to enhance the anticancer activity (Fig 2). However, the enhanced activity is related to the introduced way of rhamnose and the substituted position of rhamnose hydroxyl by acetyl groups. If only one rhamnose was introduced into emodin, the solubility of derivative was improved, but the anticancer activity was poor (S-3). Furthermore, when the hydroxyl of rhamnose were disubstituted by two acetyl groups. The anticancer activity of the generated derivative from 3, 4-hydroxyl of rhamnose disubstituted by the adjacent acetyl, is superior to that of 2, 3-hydroxyl or 2, 4-hydroxyl disubstituted derivative, that is, the former has a strong antiprolifertion effect (S-5). When emodin rhamnopyranosides derivative had its sugar chains prolonged, the anticancer activity in vitro was significantly enhanced (S-8). Similarly, when the hydroxyl groups of C-1 and C-8 position of emodin are methylated, its anticancer activity also was improved (S-9).

### Inhibitory effect of EM-d-Rha on multiple cancer cell lines

To confirm the inhibitory effect and antiproliferative activity of EM-d-Rha on cancer cells in vitro, and understand the dose-response relationship, we further conducted the experiments on various concentrations of EM-d-Rha against multiple cancer cells. The IC_50_ values were calculated from these data.

As shown in Table 2 and Table 3, we can see that EM-d-Rha has very significant anti-proliferative effects against various cancer cell lines in a concentration-dependent manner. However, its half-inhibitory concentration values to normal tissue cells are higher than to cancer cells. It is possible that the growth inhibitory effects of EM-d-Rha on cells are selective to some extent.

**Table 2:** IC50 values of EM-d-Rha against different cell lines.

Tissue Sources	Cell lines	IC_50_ for EM-d-Rha (μM)
Human hepatic carcinoma	HepG2	1.81±0.44
Human cervical cancer	HeLa	1.51±0.31
Human ovarian cancer	OVCAR-3	2.26±0.92
Human lung cancer	A549	1.75±0.63
Human umbilical vein endothelial	HUVEC	18.73±3.90
Human normal liver	L02	19.70±1.70
Human breast cancer	MCF-7	8.56±1.04
Human chronic myeloid leukemia	K562	2.80±0.11
Human gastric cancer	SGC-7901	1.99±0.12

Cancer cells treated with different concentrations of EM-d-Rha were assayed for cell viability by MTT method at 72h and IC_50_ values were calculated using GraphPad Prism 5 software. IC_50_ values for EM-d-Rha against each cell lines are means of three independent experiments (n = 3, mean±S.E.M).

**Table 3:** Inhibition rate of EM-d-Rha against various cancer cells lines.

Cancer cell lines	The inhibition rate of EM-d-Rha (%)				
	0.1μM	0.5μM	2.5μM	12.5μM	62.5μM
HepG2	4.60±5.04	24.26±6.54	70.69±3.54	90.97±0.72	91.46±0.46
HeLa	14.81±5.61	37.62±2.81	49.17±3.78	69.08±3.36	89.74±0.63
OVCAR-3	1.40±0.21	2.95±1.12	66.04±1.27	83.64±1.15	90.64±0.74
A549	13.95±0.02	27.26±2.19	33.73±1.46	63.52±1.03	75.96±0.58
MCF-7	16.79±1.40	20.14±3.83	41.40±6.53	62.67±6.81	77.07±5.10
SCG-7901	20.10±0.18	40.99±0.02	42.63±0.02	95.01±0.01	94.33±0.05
K562	0	15.14±1.13	45.86±0.11	78.74±0.01	80.64±0.03

Cancer cells treated with different concentrations of EM-d-Rha were assayed for cell viability by MTT method at 72h and the inhibition percentage was calculated by normalizing the absorbance of treated samples against the control. The inhibition rate of EM-d-Rha are means of three independent experiments (n = 3, mean±S.E.M).

### Dose–response curve of EM-d-Rha against HepG2

**Fig 3:**
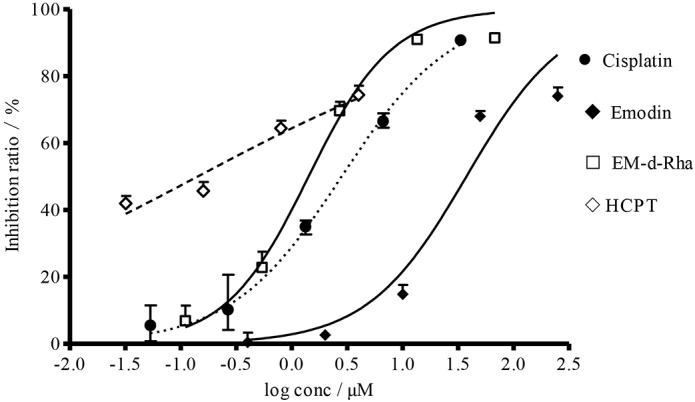
Dose-response curves of various compounds when HepG2 cells were treated for three day. Four curves represent the dose-response curve of HCPT, EM-d-Rha, Cisplatin, and Emodin against HepG2 cell from left to right in turn. The error bars represent mean± standard deviation (three replicates). The horizontal axis represents the log concentration of EM-d-Rha, the vertical axis represents the inhibition percentage.

EM-d-Rha exerted a significant anti-proliferative effect on HepG2 cells, the results led us to further investigate and compare the dose-response curves of EM-d-Rha with that of emodin, Cisplatin and HCPT. As shown in Fig 3, the growth inhibitory effect of EM-d-Rha on HepG2 cells exhibited a dose-dependent manner. With the increasing of log concentration of EM-d-Rha, the inhibition percentage of HepG2 cells significantly increased (Fig 3). Moreover, the activity of EM-d-Rha was stronger in comparison to that of emodin against HepG2 cells. However, it is inferior as compared to that of Cisplatin and HCPT against HepG2 cells.

### Effect of EM-d-Rha on the morphology of HepG2 cells

**Fig 4:**
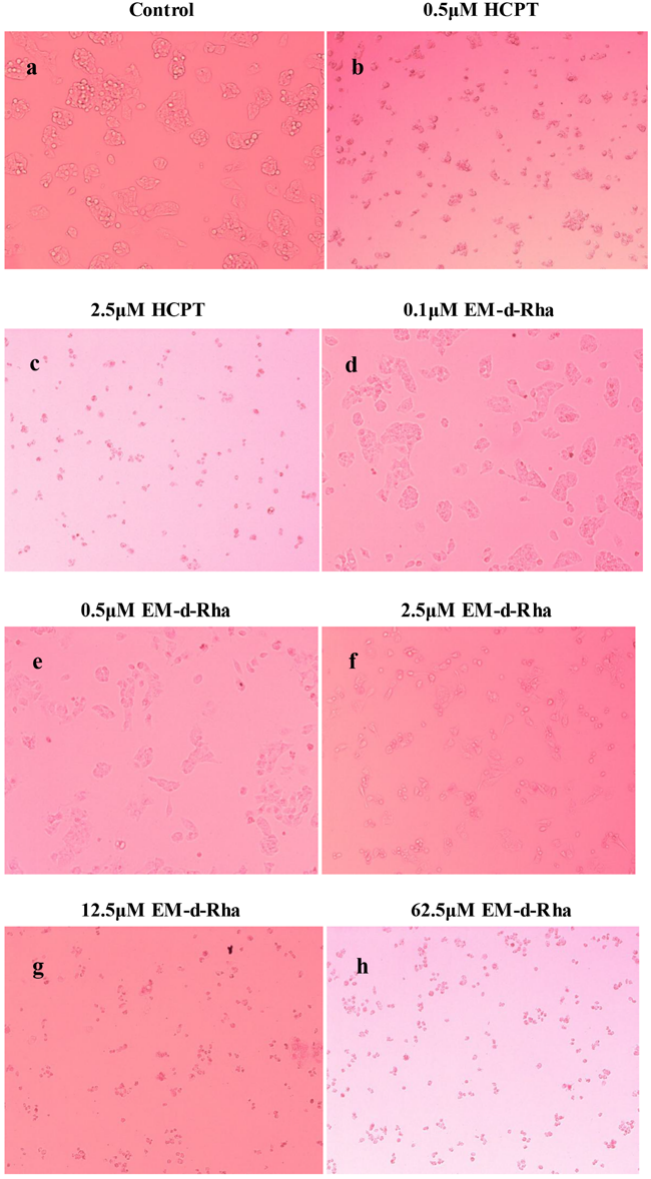
Effects of EM-d-Rha on HepG2 cell morphology. Bright-field microscopy images after incubation with HCPT for 48h at 0.5μM; 2.5μM and with EM-d-Rha for 48 h at varying concentrations: 0μM (control); 0.1μM; 0.5μM; 2.5μM; 12.5μM; 62.5μM (original magnification 100×).

To see directly the growth inhibitory effect of EM-d-Rha on HepG2 cells, we observed the morphological changes of HepG2 cells after treated (Fig 4). The morphological changes of cells may reflect the growth and death state. As a result, the untreated HepG2 cells of control group showed a high confluence of monolayer cells and flourished in the logarithmic growth phase, which had obvious morphological characteristics of eugenic cells such as intercellular tight connections, clear membrane, full cytoplasm and good refractive performance (Fig 4A). In contrast, the EM-d-Rha-treated cells clearly showed the morphological changes of apoptosis such as reduction in cell volume and losing cell-cell contacts in a dose dependent manner (Fig 4D–4H). When treated with 12.5μM EM-d-Rha, the morphological changes of HepG2 cells showed similar features to that of 2.5μM HPLC-treated (Fig 4C).

To further investigate whether the viability decrease of HepG2 cell treated with EM-d-Rha is due to apoptosis induction, we carried out the morphological observation with fluorescence microscopy which has enhanced sensitivity and contrast. HepG2 cells were stained with a DNA fluorescent dye Hoechst 33342, which has high sensitivity and low cytotoxicity. It can penetrate cancer cells membrane, and bind DNA and emit a strong indigo fluorescence. Hoechst binds DNA at the groove regions that contain generous AT base sequence. It has good water-soluble and stability. Therefore, fluorescence microscopy combines highest sensitivity with molecular specificity and exceptional image contrast. In order to verify whether the test is successful or not, we establish HCPT compound as positive control.

**Fig 5:**
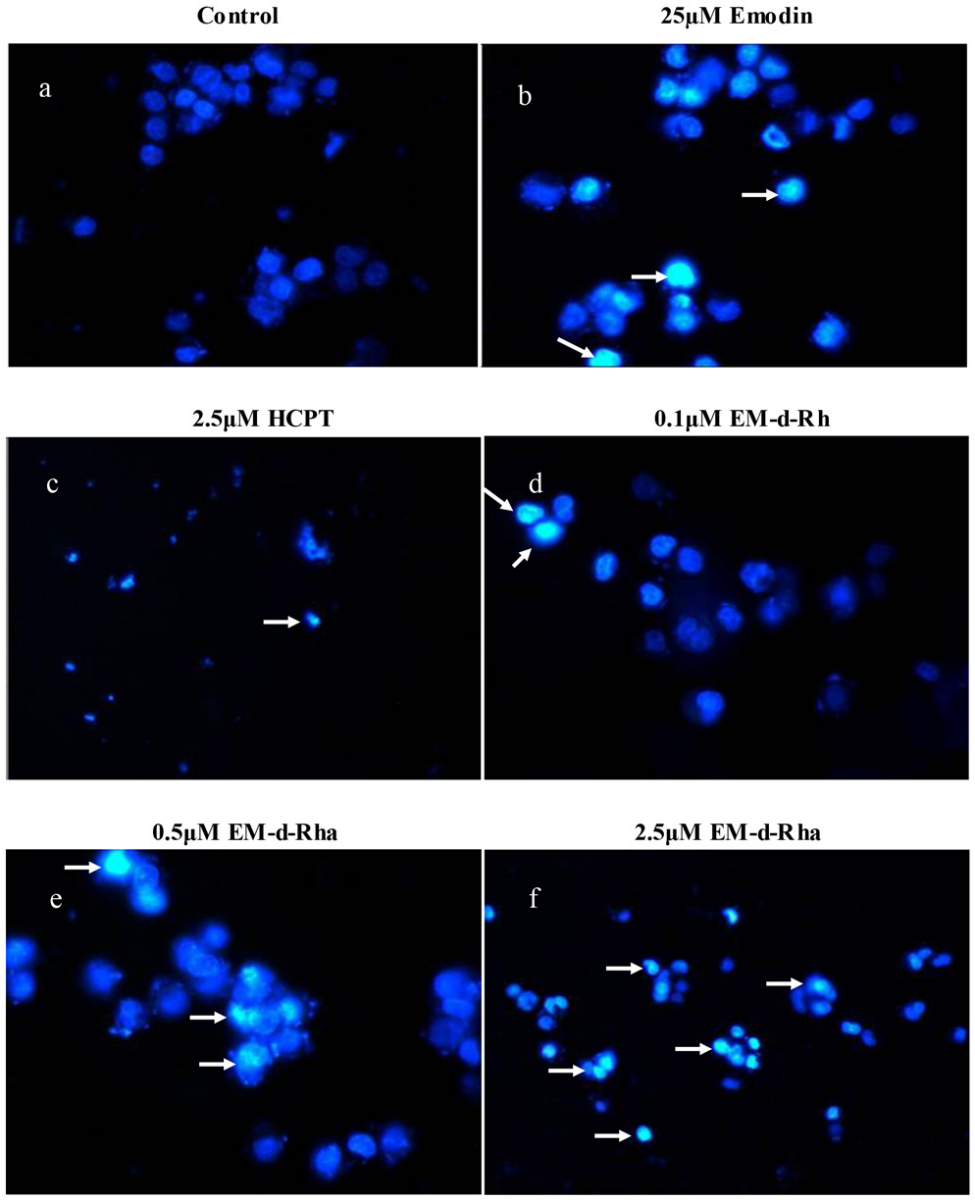
Fluorescence microscopic appearance of Hoechst 33342-stained nuclei of HepG2 cancer cell treated. The nuclei morphological changes of HepG2 cell were observed with fluorescence microscopy after incubated with 25.0μM emodin, 2.5μM HCPT for 48 h and with EM-d-Rha for 48 h at varying concentrations: 0μM (control); 0.1μM; 0.5μM; 2.5μM (original magnification 400×). The condensed and fragmented fluorescent nuclei (white arrows) of apoptotic cells are visible in the treated cells, but not in the control cells.

As shown in Fig 5, HepG2 cells showed the typical apoptotic nuclear morphology after exposure to 2.5μM HCPT for 48h, and presented cell-to-cell contact loss, nuclear shrinkage, membrane blobbing, DNA condensation and fragmentation (Fig 5C). Similarly, when HepG2 cells were treated with varying concentrations of EM-d-Rha for 48h, they clearly showed the characteristics of nuclear shrinkage and DNA condensation, and presented a dosage-dependent aggravated damage (Fig 5D–5F).Some of HepG2 cells detached from the monolayer and floated on the culture medium, however the apoptotic nuclear appearance was not observed in untreated control, HepG2 cells of the control group appeared uniform blue fluorescence (Fig 5A). HepG2 cells treated with 25μM emodin also displayed apoptotic morphological changes (Fig 5B).

### Effects of EM-d-Rha on DNA fragmentation of HepG2 cells

**Fig 6:**
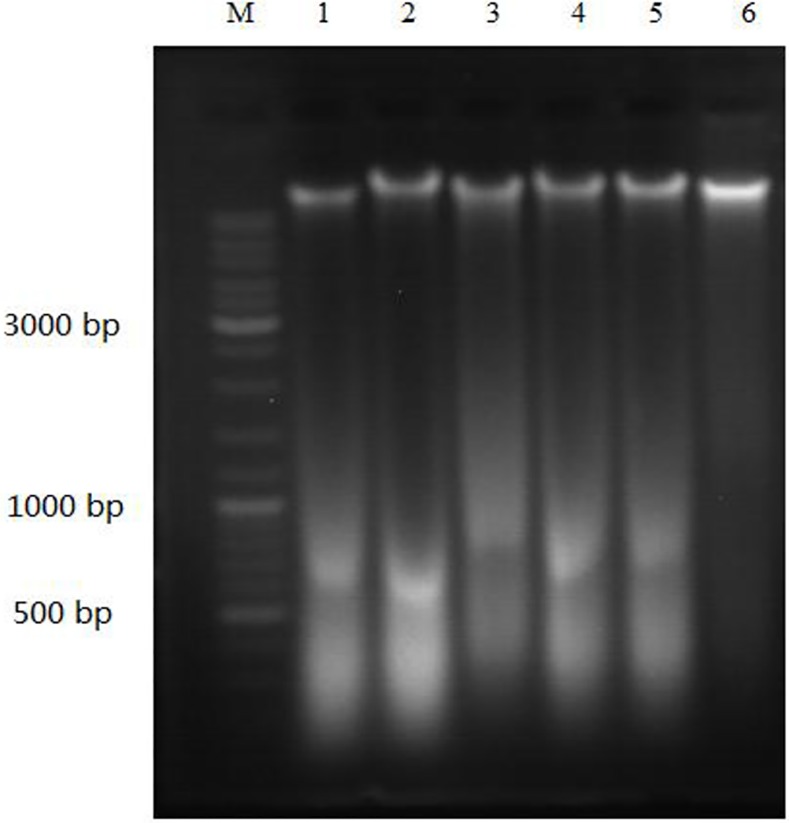
Fragmentation analysis by agarose gel electrophoresis. Lane M: DNA Ladder; Lane 6: Control; Lanes1, and 2: Cells treated with 3.0μM and 6.0μM Cisplatin; Lanes 3, 4 and 5: Cells treated with 2.5μM, 5.0μM and 10.0μM EM-d-Rha, respectively.

To further investigate whether EM-d-Rha led to DNA fragmentation of HepG2 cells, which is an important feature of cell apoptosis. The nuclear DNA was detected with agarose gel electrophoresis method. We also found that EM-d-Rha may exert its apoptotic induction effects, when HepG2 cells were treated with EM-d-Rha, the extracted DNA of HepG2 cells appeared typical ladder or fragmentation shape(Fig 6), was similar to what the DNA of HepG2 cells treated with Cisplatin presented, however, was different with the DNA shape of the untreated HepG2 cells (Fig 6). The result suggested that EM-d-Rha caused double stranded DNA breakage of HepG2 cells and induced apoptosis in HepG2 cells.

### Apoptosis induction effects of EM-d-Rha on HepG2 and OVCAR-3 cells

**Fig 7:**
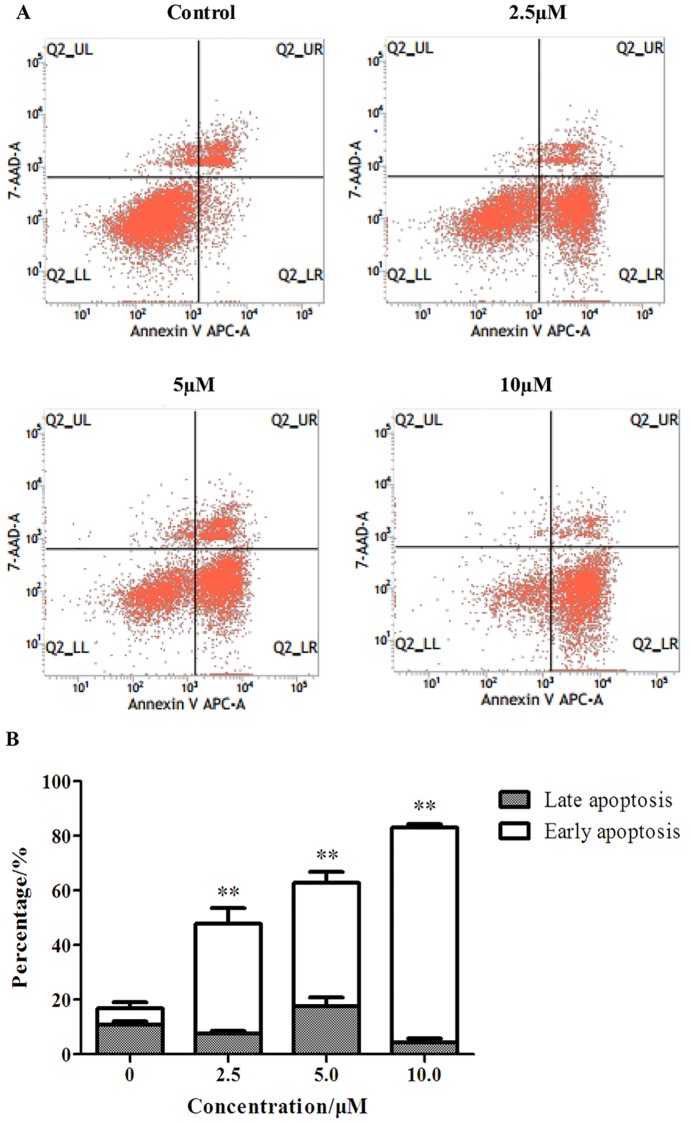
Flow cytometry analysis of apoptosis of HepG2 cells treated with EM-d-Rha. HepG2 cells were incubated for 72h with EM-d-Rha at 0, 2.5μM, 5μM, 10μM, respectively. And cells were stained with FITC conjugated Annexin V and 7-AAD. A: Representative dot plots of Annexin V-FITC/7-AAD staining. a: control, 72h; b: 2.5μM EM-d-Rha, 72h; c: 5μM EM-d-Rha, 72h; d: 10μM EM-d-Rha, 72h. In the scatter plot of double variable flow cytometry, the lower left quadrant Q2-LL shows the living cells (double negative, Annexin V-APC ^-^/7-AAD^-^) population. The lower right quadrant Q2-LR represents the early apoptotic (Annexin V-APC ^+^/7-AAD^-^) population and upper right quadrant Q2-UR stands for the late apoptotic/necrotic (Annexin V-APC ^+^/7-AAD^+^) population; B: Data pooled from three independent experiments show the percentage of apoptotic cells. Difference was considered statistically significant when *p<0.05 and **p<0.01 *vs* control group.

**Fig 8:**
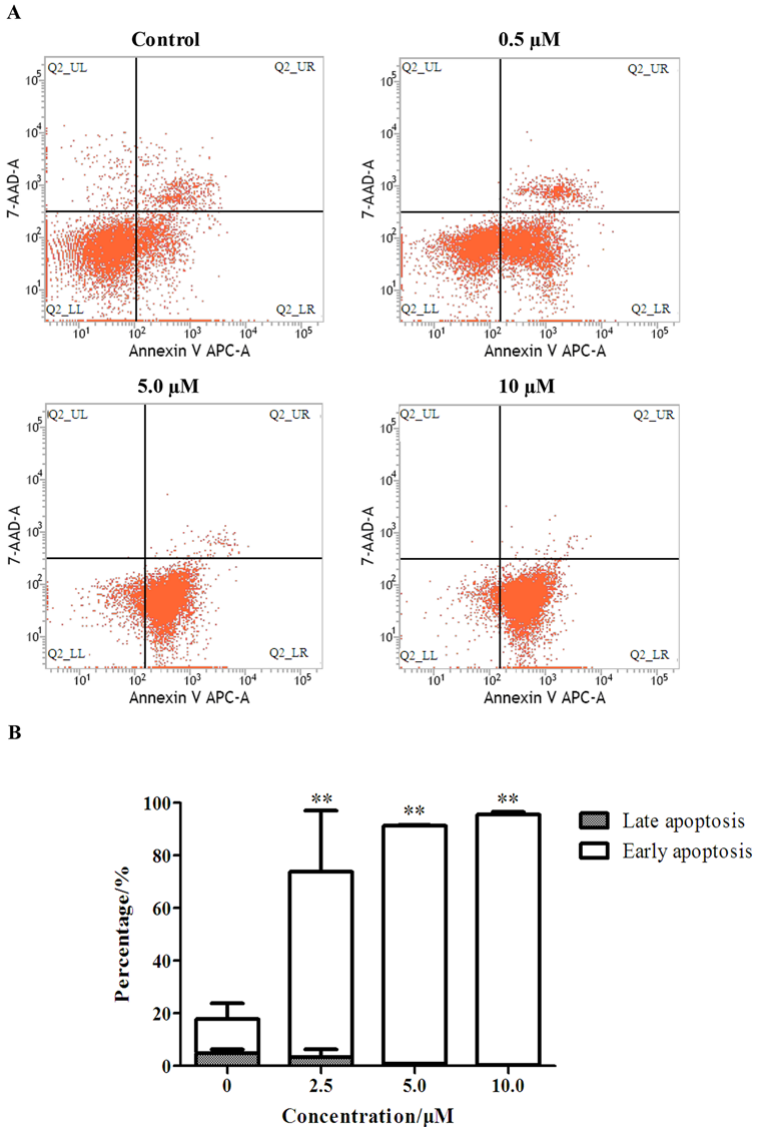
Flow cytometry analysis of apoptosis of OVCAR-3 cells treated with EM-d-Rha. OVCAR-3 cells were incubated for 72h with EM-d-Rha at 0, 2.5μM, 5μM, 10μM, respectively. And cells were stained with FITC conjugated Annexin V and 7-AAD. A: Representative dot plots of Annexin V-FITC/7-AAD staining. a: control, 72h; b: 2.5μM EM-d-Rha, 72h; c: 5μM EM-d-Rha, 72h; d: 10μM EM-d-Rha, 72h. B: Data pooled from three independent experiments show the percentage of apoptotic cells. Difference was considered statistically significant when *p<0.05 and **p<0.01 *vs control group*.

To demonstrated further that EM-d-Rha may inhibit the growth and proliferation of HepG2 cells and OVCAR-3 cells via the mechanism of apoptosis induction, we also applied flow cytometry analysis with the double staining method of Annexin V-APC and 7-AAD (7-amino-actinomycin D). The apoptosis induction activity of S-8 was detected by the bind between Annexin V and phosphatidylserine (PS) which mainly distribute in the cytoplasmic side of living cells, however, during the early stage of cell apoptosis, PS emigrate from the cytoplasmic side to the cell wall side, so this can generate specific bind between Annexin V and PS. 7-AAD is a nucleic acid stain, which can permeate the cell membranes at the late stage of cell apoptosis and the dead cells and further dye their nucleus red. So the combined use of Annexin V-APC and 7-AAD can distinguish late apoptotic cells from early apoptotic cells. HepG2 cells and OVCAR-3 cells underwent apoptosis after exposure to EM-d-Rha at 2.5μM, 5μM and 10μM for 48h (Fig 7 and Fig 8). The percentage of apoptotic cells is shown in Table 4 and Table 5.

**Table 4:** The apoptosis rates of HepG2 treated with different concentration EM-d-Rha.

Groups	Early apoptosis/%	Late apoptosis/%	Living cell/%
Control group	6.16±2.20	10.71±1.42	79.87±1.84
2.5μM EM-d-Rha group	40.40±5.70**	7.50±1.04	51.20±5.10**
5μM EM-d-Rha group	45.20±4.17**	17.53±3.16	36.40±3.23**
10μM EM-d-Rha group	78.77±1.22**	4.22±1.63	16.53±0.68**

The apoptosis rates of HepG2 cells are means of three independent experiments (n = 3, mean ±S.E.M).

**represent *p<0*.*01 vs*. *control group*.

**Table 5:** The apoptosis rates of OVCAR-3 cells treated with different concentration EM-d-Rha.

Groups	Early apoptosis/%	Late apoptosis/%	Living cell/%
Control group	13.01±5.81	4.87±1.42	81.36±8.14
2.5μM EM-d-Rha group	70.60±23.06**	3.30±2.95	25.82±20.14**
5μM EM-d-Rha group	90.52±0.20**	0.81±0.05*	8.60±0.19**
10μM EM-d-Rha group	95.09±1.03**	0.35±0.06**	4.53±0.99**

The apoptosis rates of OVCAR-3 cells are means of three independent experiments (n = 3, mean±S.E.M).

*represent *p<0*.*05 vs*. *control group*

**represent *p<0*.*01 vs*. *control group*.

EM-d-Rha may significantly induce HepG2 cells and OVCAR-3 cells apoptosis in the early growth stage (Fig 7 and Fig 8). We can see from Table 5, when OVCAR-3 cells treated with 10μM EM-d-Rha, the early apoptosis rate of OVCAR-3 cells reached to 95.09%, and the living cells only remained 4.53%. Similarly, when HepG2 cells treated with 10μM EM-d-Rha, the early apoptosis rate reach to 78.77%, and the living cells only account for 16.53% (Table 4).

### Effect on cell cycle distribution

**Fig 9:**
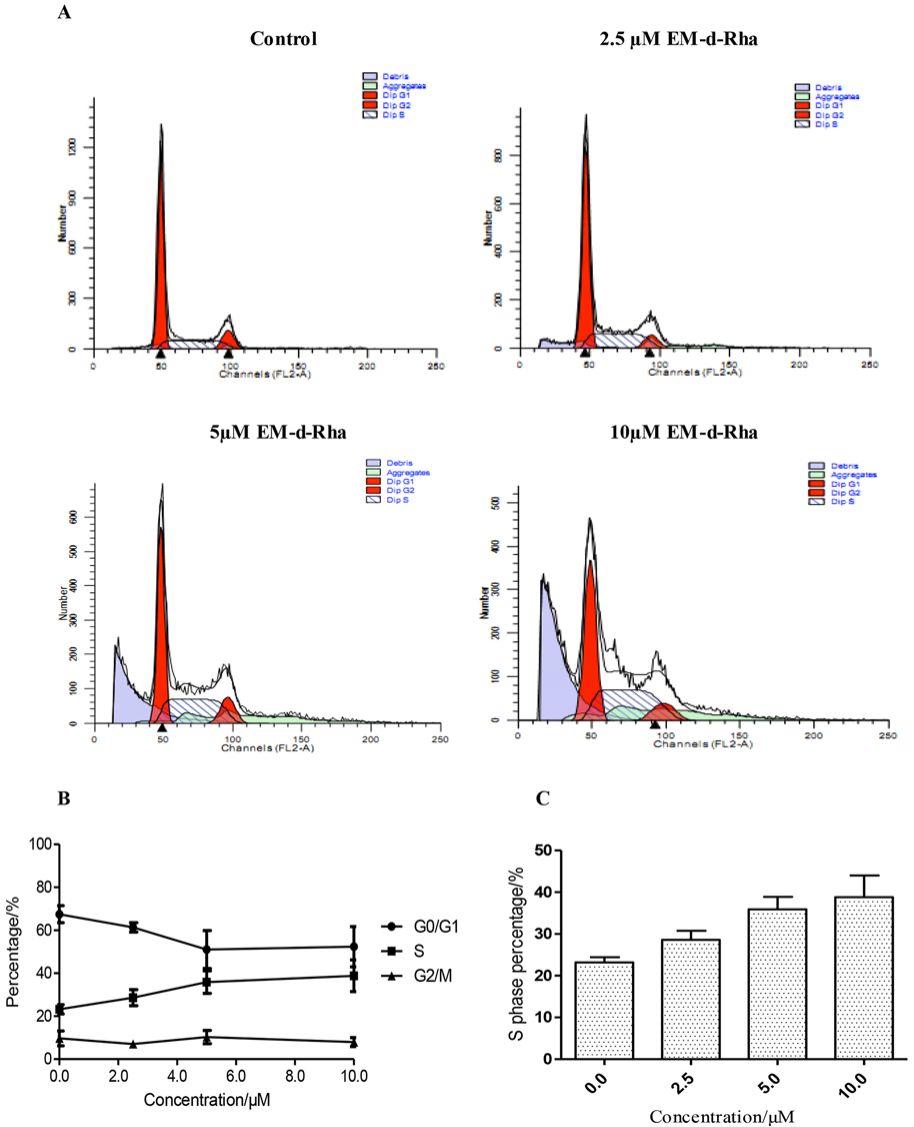
Effects of EM-d-Rha on HepG2 cell cycle distribution *in vitro*. After HepG2 cell exposure to 0μM, 2.5μM, 5.0μM and 10μM EM-d-Rha for 48h, cells were harvested and stained by propidium iodide, then cell cycle distribution was examined by FACS flow cytometric analysis (A). Data pooled from three independent experiments show the percentage of cell cycle distribution of HepG2 cells (B). The percentage of S phase over total cells was calculated and expressed on each histogram (C).The error bars represent mean± standard deviation (B and C). Difference was considered statistically significant when *p<0.05 *vs*. control group.

Cell cycle regulation was important for cell proliferation, so cell cycle arrest was the reason of cell apoptosis induced by anticancer agents. To explore whether the antiproliferative effect of EM-d-Rha was related to cell cycle arrest, the cell cycle distribution was detected by flow cytometry with the Propidium Iodine (PI) stain method. As shown in Fig 9, the untreated control group resulted in an accumulation of cells in G1, S and G2/M phase by 67.48%, 23.19% and 9.68% respectively, so the cell cycle of control group mainly arrested in G1 phase. However, after HepG2 cells exposure to various concentration EM-d-Rha(2.5μM, 5.0μM, and 10μM) for 48h, EM-d-Rha affected cell cycle distribution, leading to cell cycle arrest at S phase, cell amount at S phase increased from 23.19%(control group) to 28.59%((2.5μM), 35.88%(5.0μM) and 38.83%(10μM) respectively (Fig 9 and Table 6). On the contrary, there was a slight decrease in the number of cells in G0/G1 phase. S phase cells significantly increased in a dose-dependent manner. The results suggested that the growth inhibition effect of EM-d-Rha on HepG2 cell was related to cell cycle arrest at the S phase.

**Table 6:** Percentage of cell cycle distribution in HepG2 treated with EM-d-Rha.

Groups	G0/G1 (%)	S (%)	G2/M (%)
Control group	67.48±3.94%	23.19±2.18%	9.68±3.46%
2.5μM EM-d-Rha	61.37±2.16%	28.59±3.75%	7.01±1.73%
5.0μM EM-d-Rha	51.03±8.84%*	35.88±5.25%*	10.31±3.08%
10.0μM EM-d-Rha	52.35±9.37%*	38.83±7.35%*	7.96±2.07%

Percentage of cell cycle distribution are means of three independent experiments (n = 3, mean ±S.E.M).

*represent *p<0*.*05 vs*. *control group*.

### Effect of EM-d-Rha induces the loss of ΔΨm in HepG2 cells

Mitochondria are an integral part of the apoptotic machinery, and the loss of mitochondrial transmembrane potential (ΔΨm) is a classical evidence for apoptosis. To further study whether the mitochondria events were involved in the caspase-dependent apotosis induction of EM-d-Rha, ΔΨm was detected by flow cytometry with staining method of fluorescent dye Rhodamine123. Rhodamine123 is a positive-ion fluorescent dye and indicator of ΔΨm. By dependent on the ΔΨm of living cells, Rhodamine123 can pass through cell membrane and specifically gather in the mitochondria. So the absorption values of Rhodamine123 by cell mitochondria can reflect the changes of ΔΨm. When cell apoptosis happened, the integrity of mitochondrial membrane was destroyed and ΔΨm collapsed, so the ΔΨm of cells decrease or loss. Moreover, the apoptosis occurrence and the changes of ΔΨm can be detected through the fluorescence signal intensity of Rhodamine123 with flow cytometry. In this study, the detection wavelengths include excitation and emission wavelength was adjusted to 480nm and 520nm, respectively.

**Fig 10:**
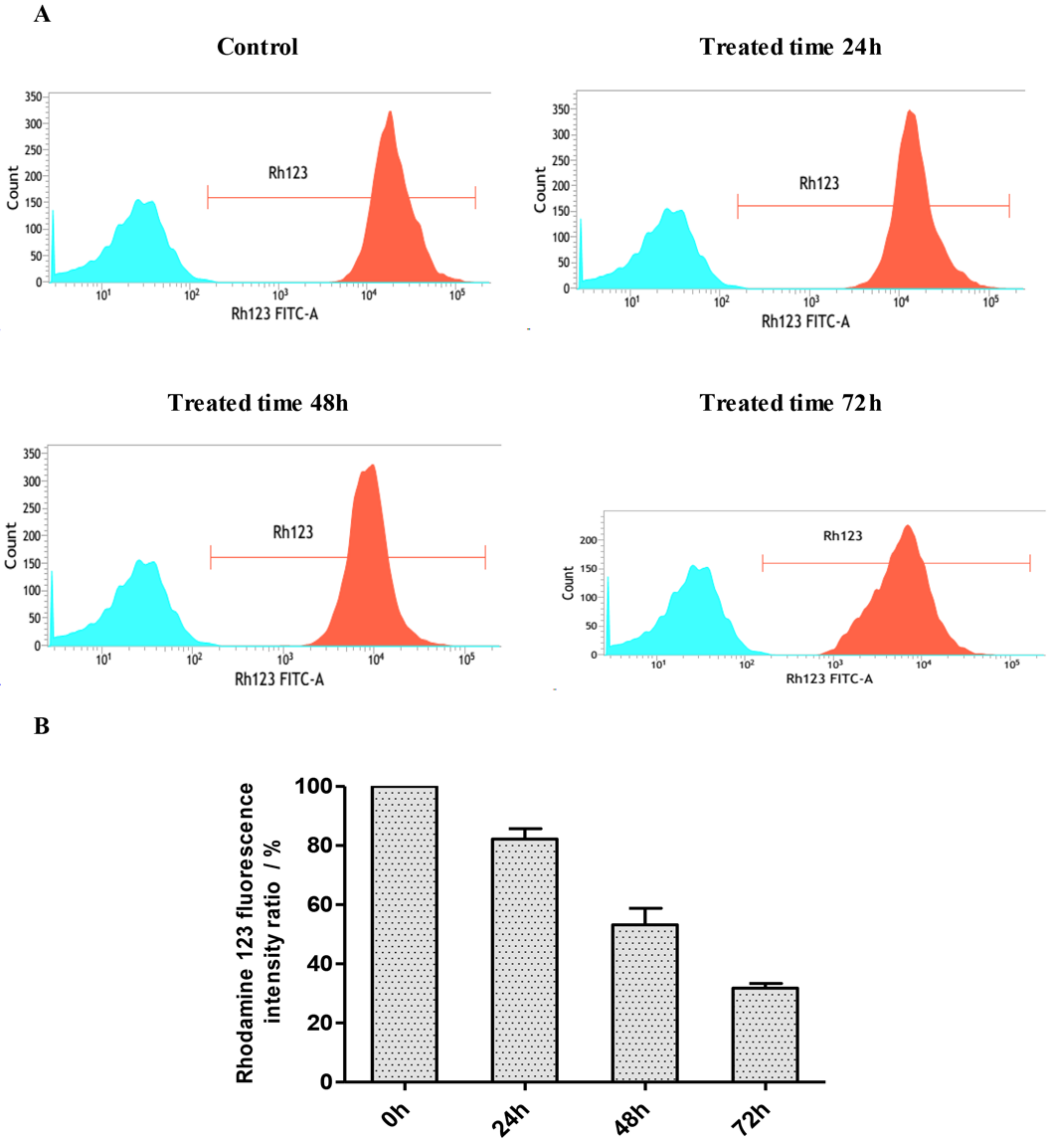
Effects of EM-d-Rha on HepG2 mitochondrial membrane potential (ΔΨm) in different treated times. ΔΨm was detected by flow cytometry. A: HepG2 cells were incubated with 5μM EM-d-Rha for 0, 24h, 48h and 72h respectively. B: Data pooled from three replicates show the percentage of HepG2 ΔΨm *vs*. control group. The error bars represent mean± standard deviation. Difference was considered statistically significant when *p<0.05 and **p<0.01 *vs*. *control group*.

The photograph of ΔΨm can be seen from Fig 10A, in short treated time, the ΔΨm peak of HepG2 cells moved left along horizontal ordinate, it means ΔΨm decrease. When treated for a long time, the peak height and peak area of ΔΨm declined besides left movement of the ΔΨm peak. The results indicated that ΔΨm decreased in a time-dependent manner (Fig 10). The relative mean fluorescence intensity of ΔΨm of HepG2 cells decreased to 31.77±2.84% of that of the control group after treated with 5.0μM EM-d-Rha for 72h (Table 7). So the present study demonstrated that the anti-proliferative and growth inhibition effects of EM-d-Rha mainly associated with apoptosis induction, which led to mitochondrial dysfunction and the loss of ΔΨm.

**Table 7:** Percentage of mitochondria membrane potential (ΔΨm) in HepG2 treated with EM-d-Rha in different times.

Groups	Time treated (h)	Percentage of ΔΨm (*Vs*. *control group*) (%)
Control group	0	100.00±0.00
5.0μM EM-d-Rha	24	82.22±5.97*
5.0μM EM-d-Rha	48	53.21±9.71*
5.0μM EM-d-Rha	72	31.77±2.84**

Percentage of ΔΨm are means of three independent experiments (n = 3, mean±S.E.M).

*Vs*. *control group*

*represent *p<0*.*05*

**represent *p<0*.*01*

### EM-d-Rha upregulating the expression of apoptosis relative genes

**Fig 11:**
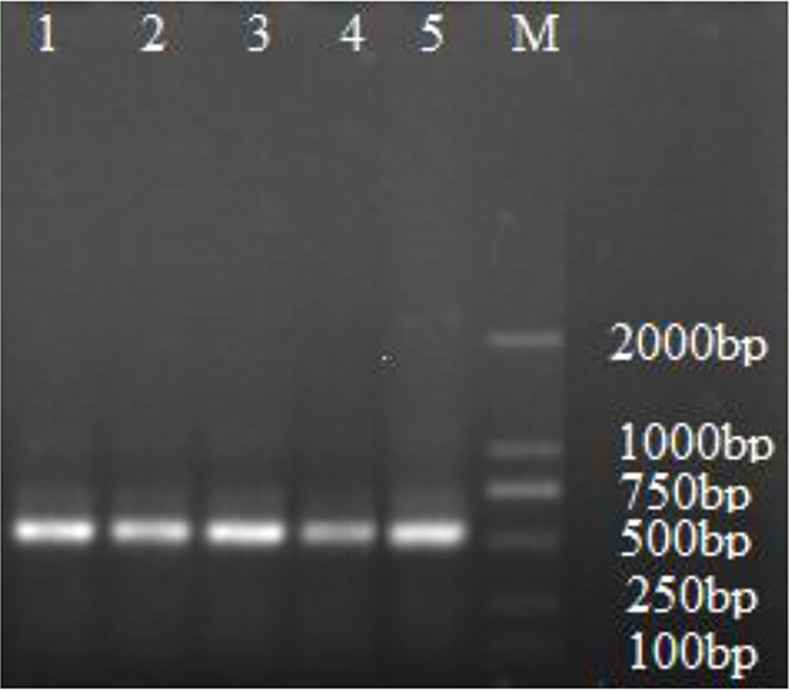
β-actin gene analysis of cDNA template by agarose gel electrophoresis. Lane M: DNA Ladder; Lane 1: Control; Lanes2~5: cDNA of the sample treated with 0.70μM, 1.40μM, 2.80μM and 5.60μM EM-d-Rha, respectively.

RNA of all groups was extracted and the amount was measured by the described previously. In this study, the A_260_/A_280_ ratio values of the extracted RNA located in the range of 2.0~2.1 region. So the RNA purity of all samples is very high and suitable for further analysis. Additionally, the templates efficiency was checked by agarose gel electrophoresis analysis, the results suggested that the single and same size β-actin gene band (549bp) can be amplified using the synthesized cDNA products as templates (Fig 11), so the quality of the synthesized cDNA products was good as template for RT-PCR reaction system.

**Fig 12:**
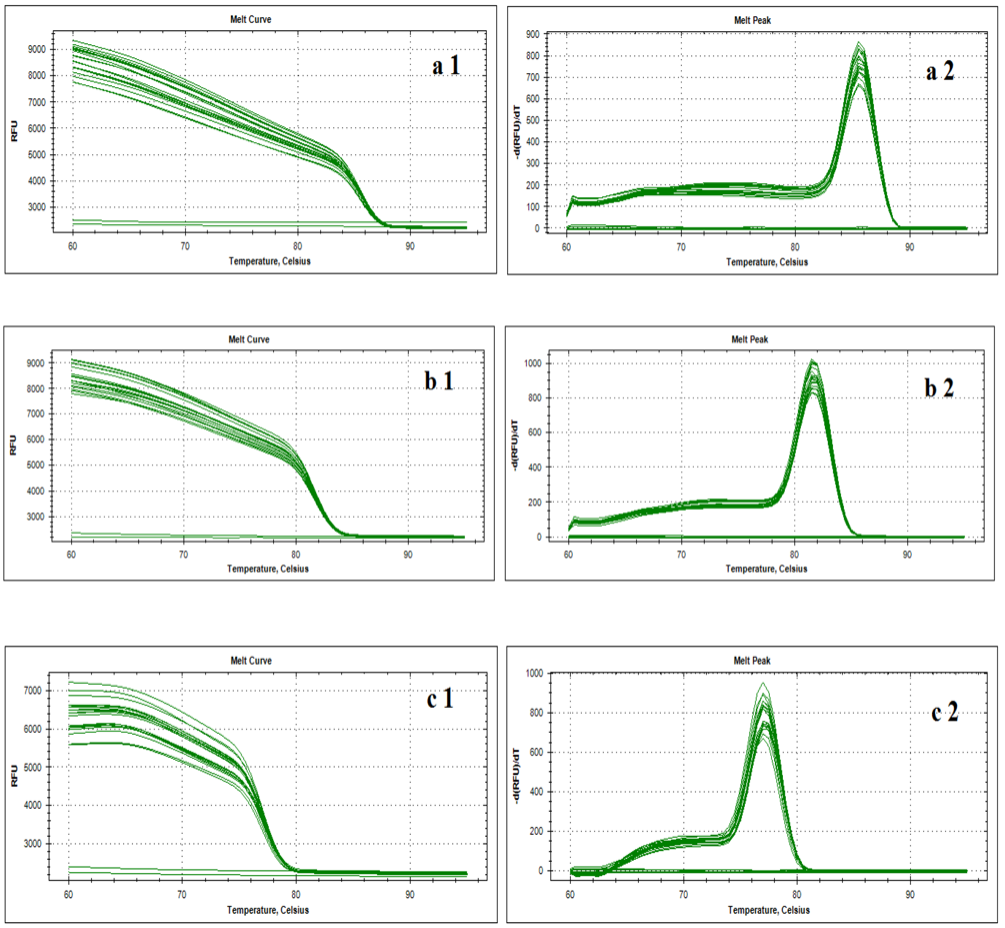
Different gene RT-PCR product melt curve analysis. a1and a2 represent the melt curve and peak of β-actin gene, respectively; b1 and b2 represent the melt curve and peak of Cyto C gene, respectively; c1 and c2 represent the melt curve and peak of caspase-3, respectively. For the genes β-actin, Cyto C and caspase-3 with corresponding melt temperatures of the specific target amplicons.

The RT-qPCR assay results indicated that the melt curve of Cyto C, Caspase-3 and internal reference gene β-actin products had a single and sharp peak respectively, and melt temperature was uniform (Fig 12). No extra shape waves appeared in the curves. Corresponding melt temperature showed Cyto C: 85.50°C, Caspase-3: 81.50°C and β-actin: 77.00°C. The data suggested that the amplification products of Cyto C, Caspase-3 and β-actin gene were specific, and the performance of the respective primer pairs was efficient.

**Fig 13:**
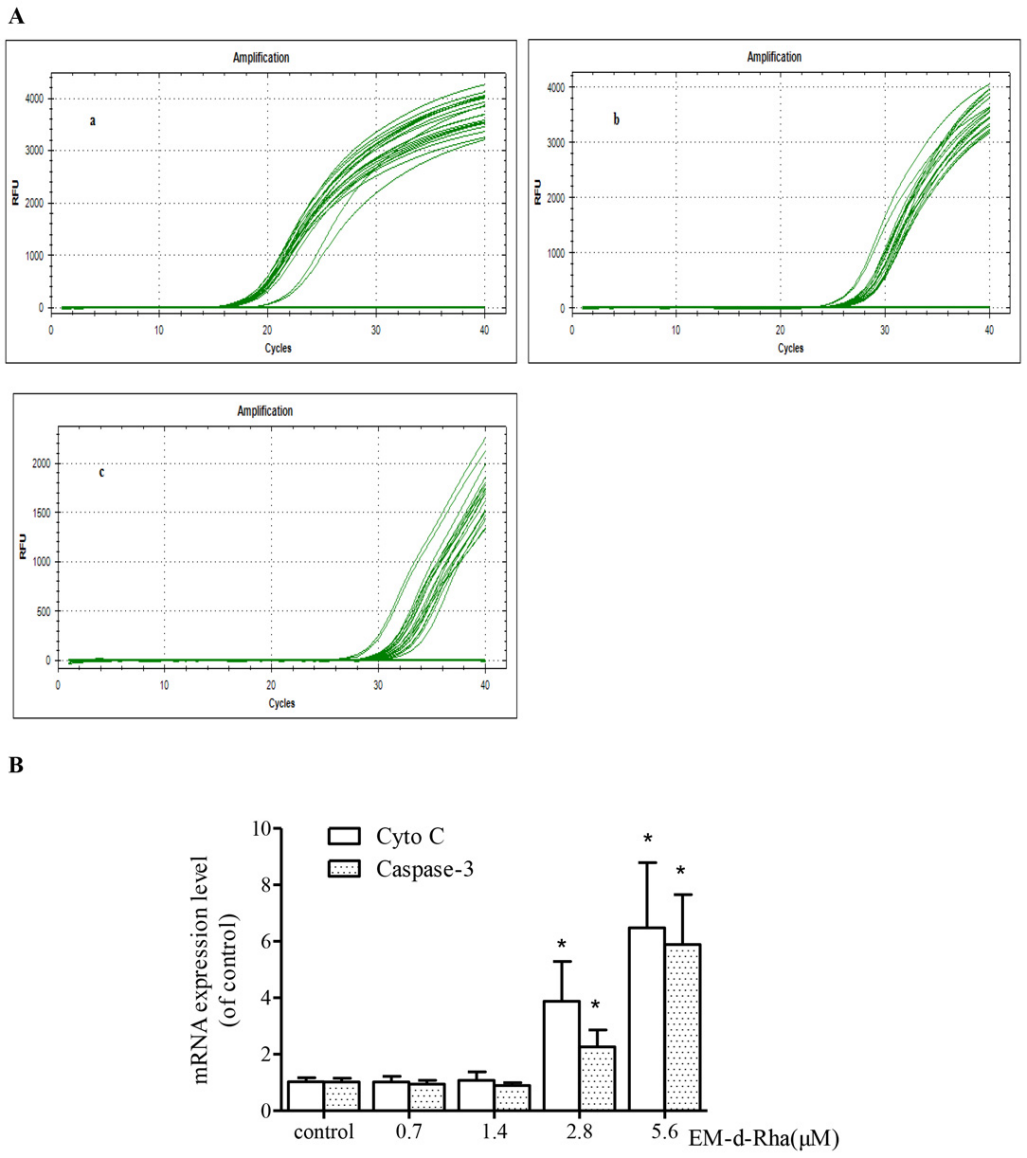
Effects of EM-d-Rha regulating the expression of Cytochrome C and Caspase-3. The gene expression levels of Cyto C and Caspase-3 were quantified by detecting the threshold cycle (CT) values with real-time quantitative PCR. A: Real time quantitative PCR amplification curve for different gene, a, b and c represents β-actin, cyto C and caspase-3, respectively.B: The mean fold change of cyto C and Caspase-3 expression is calculated by the 2^-Δ ΔCT^ method and expressed on each histogram. The values represent means ±S.E of four replicates experiments. Asterisk represents significantly different when compared to control (*p<0*.*05*).

The mRNA expression levels of Cyto C and Caspase-3 in HepG2 cells treated with EM-d-Rha showed a significant up-regulating when compared to the control group (*p<0*.*05*)(Fig 13 and Table 8). Moreover, the up-regulated gene expression presented a concentration-dependent manner. Obviously, when the treatment concentration of EM-d-Rha reached to 2.80μM, the mRNA expression level of Cyto C and Caspase-3 increased dramatically. The present study suggested that the up-regulating expression of Cyto C and Caspase-3 may be involved in the processes of EM-d-Rha-induced HepG2 cells apoptosis.

**Table 8:** Effects of EM-d-Rha regulating Cyto C and Caspase-3 gene expression of HepG2 cells.

Groups	Caspase-3	Cytochrome C
Control group	1.022±0.258	1.030±0.280
0.70μM EM-d-Rha	0.949±0.259	1.009±0.475
1.40μM EM-d-Rha	0.900±0.190	1.086±0.727
2.80μM EM-d-Rha	2.727±0.715*	3.258±2.402*
5.60μM EM-d-Rha	4.413±3.730*	5.625±4.950*

All experiments were performed in duplicate, nomalized either to caspase-3 or Cytochrome C, and each value was reported as level relative to controls. Beta-actin was used as an internal control. The fold change for mRNA expression level was calculated using the ΔCt method as described in materials and methods. Data are expressed as means of four independent experiments (n = 4, mean±S.E.M).

*represent *p<0*.*05 vs*. control group.

### EM-d-Rha regulating apoptotic protein expressions in HepG2 cells

**Fig 14:**
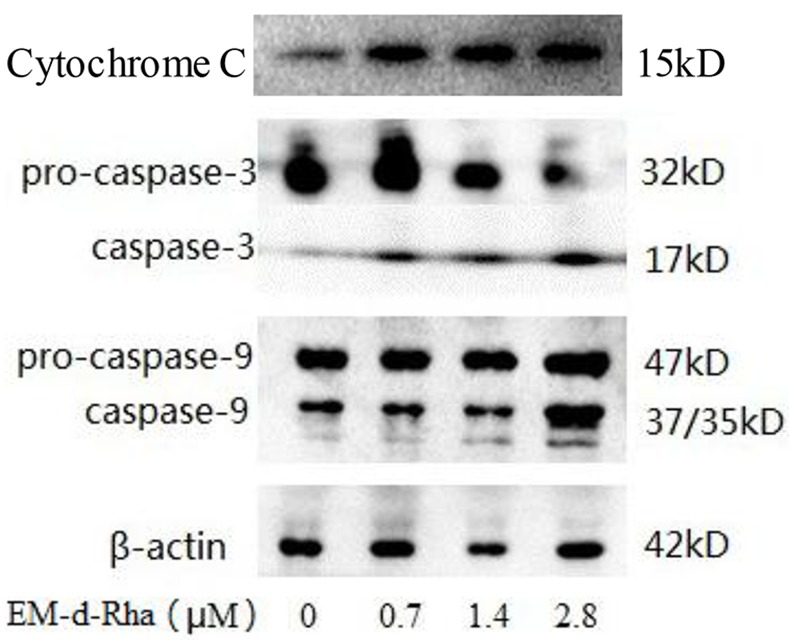
The effect of EM-d-Rha up-regulating the expression level of the proteins Cyto C, caspase-3 and caspase-9. Western blot analysis for Cyto C, Caspase-3 and Caspase-9 was performed with specific antibodies and β-actin as reference protein. The activation of caspase-3 and -9, which can be observed by the same molecular weight protein band 17kD and 37/35kD in Western blot.

Cysteine aspartic acid specific protease abbreviated as Caspase. Caspase-3 and -9 belong to the family of cysteine proteases that play a critical role in the process of cells apoptosis. However, caspase-3 and -9 are synthesized by cells and initially present in the inactive state, so they are also called pro-caspase-3 and pro-caspase-9 respectively. Only after the specific site of proteins are cleaved and become active, they can perform cell apoptosis function. To explore whether EM-d-Rha achieve the apoptosis induction action through mediating the protein expression of caspase-3 and caspase-9, the expression levels of Cyto C, caspase-3 and caspase-9 proteins were detected with western-blot. The results indicated that the three proteins expression increased at a concentration-dependent manner (Fig 14), when compared to control group. So we draw a conclusion that EM-d-Rha caused the release of cytochrome c from mitochondria, followed by the activation of caspase-9 and caspase-3, leading to apoptosis. EM-d-Rha activated intrinsic pathways related to apoptosis.

## Discussion

Emodin is known to have various therapeutic properties, such as anti-inflammatory, antibacterial, and anticancer activity. Moreover, anti-proliferative effects have been well documented in a variety of cancer cell lines.

In more recent years, extensive research has focused on developing novel emodin derivatives to improve antitumor activities. Among these attempts, that L-rhamnopyranosides is connected to planar aromatic of emodin and has the rhamnose sugar chain prolonged, meanwhile modify the rhamnosyl moiety of anthracene rhamnosides with an acetyl group, such as C-10 carbonyl moiety of emodin substituted by acetylmethylene, can help to improve and enhance the anticancer activity of emodin. In this study, we gained basic data about the structure-activity relationship of the emodin L-rhamnopyranosides derivatives, although the relationship has not been completely understood.

Further findings indicated that 3-(2”,3”-Di-O-acetyl-α-L-rhamnopyranosyl-(1→4)-2’,3’-di-O-acetyl-α-L-rhamnopyranosyl)-emodin (EM-d-Rha) in all the synthesized emodin L-rhamnopyranosides derivatives has good anticancer efficacy. EM-d-Rha, a novel anthracycline with a tricyclic planar chromophore skeleton,contains O-acetyl and α-L-rhamnopyranosyl groups, that showed obvious anti-proliferative activity and growth inhibitory effect of cancer cells. The results verified that the IC_50_ values of EM-d-Rha against various human cancer cell lines, including HepG2, HeLa, OVCAR-3, A549, MCF-7, K562 and SGC-790, locate in the range between 1.5μM and 8.56μM. Moreover, HepG2 cells morphology changed significantly after treated with EM-d-Rha for 48h. Compared with emodin, the anticancer activity of EM-d-Rha increased by more than10-fold in vitro, we found that EM-d-Rha were able to achieve the enhanced bioactivity as well as the reduced toxicity. However, the mechanism actions of inhibiting HepG2 cancer of EM-d-Rha are yet to be clear.

In order to become malignant, cancer cells must find a way to evade the apoptotic cascade, which otherwise would result in their elimination. This evasion also renders tumors resistant to apotosis-inducing cytotoxic agents. For these reasons many laboratories are researching for small molecules that can restore the blocked apoptosis in cancer cells [49]. Thus, targeting critical apoptosis regulators is a promising strategy in cancer therapy [50]. In the present study, we explored apoptosis induction action. Compared with untreated HepG2 cells, EM-d-Rha induced the loss of mitochondrial membrane potential (ΔΨm) in a time-dependent manner and broke the genomic DNA in a dose-dependent manner. Mitochondria are an integral part of the apoptotic system, and the loss of ΔΨm is a remarkable characteristic of apoptosis. Moreover, we observed the typical apoptotic nuclear changes such as loss of cell-to-cell contact, nuclear shrinkage, and DNA condensation after HepG2 cells exposure to 0.5μM EM-d-Rha for 48h. The flow cytometry analysis also confirmed that HepG2 cells and OVCAR-3 cells treated with EM-d-Rha presented early stage apoptotic signs. All results keep consistent and suggested that EM-d-Rha induced apoptosis and significantly decreased mitochondrial membrane potential in cancer cells.

Research shows that the endonucleases responsible for nuclear DNA fragmentation come from the mitochondrial intermembrane space during mitochondrial dysfunction. Nuclear DNA fragmentation can be considered an additional indirect marker of mitochondrial dysfunction.

Moreover, mitochondria contain several potentially apoptotic factors, including cytochrome C, procaspases 3 and 9. The disruption of ΔΨm leads to leakage of these proapoptotic proteins into cytosol and triggers apoptosis [51]. Interestingly, in this study the levels of apoptosis-related signals such as cytochrome C, caspase-3 and -9 were found to significantly increase. Our results show that 2.8μM EM-d-Rha upregulated mRNA expression of caspase-3 and cytochrome C level by approximately 2.7 and 3.2 fold respectively than untreated HepG2 cells. In addition, EM-d-Rha could cause cleavages of procaspase-3 and -9, and mediated the overexpression of cyto C, caspase-3 and caspase-9 protein, so EM-d-Rha exert its apoptotic induction effects via regulating the activity of caspase-9 and the expression of cleaved-caspase-3.

In conclusion, the present study demonstrates that EM-d-Rha may exerted a very significant anti-proliferative activity and growth inhibition effects on HepG2 cells and OVCAR-3 cells, and inhibited the growth and proliferation of HepG2 and OVCAR-3 cells through the pathway of apoptosis induction. According to related biochemistry analysis (e.g. mitochondrial membrane potential, DNA fragmentation analysis, and caspase gene and protein analysis, etc.), the possible molecular mechanism of EM-d-Rha apoptosis induction may mainly through the intrinsic apoptotic signal pathway, which associated with mitochondrial dysfunction. That is, EM-d-Rha caused the release of apoptosis-inducing factors and Cytochrome C from mitochondria, followed by the activation of caspase-3, which led to nuclear shrinkage and apoptosis. These findings suggested that EM-d-Rha was a more effective chemotherapeutical agent for cancer cells than emodin. Despite these encouraging results, further investigation is warranted as EM-d-Rha has been shown to modulate one or more key regulators of cancer growth.
